# Linear Morphea in the Context of Severe Acute Respiratory Syndrome Coronavirus 2 (SARS-CoV-2) Infection: A Unique Dermatological Manifestation

**DOI:** 10.7759/cureus.57031

**Published:** 2024-03-27

**Authors:** Mariam Harb, Ahmad Rached, Fouad El Sayed

**Affiliations:** 1 Dermatology, Lebanese University Faculty of Medicine, Beirut, LBN

**Keywords:** vaccination, covid-19 infection, scleroderma, linear morphea, generalized morphea

## Abstract

Morphea, a rare skin disorder characterized by localized areas of thickened and sclerotic skin, typically presents as circumscribed plaques. The linear variant, however, manifests as linear bands of sclerosis affecting the extremities, and its association with coronavirus disease 2019 (COVID-19) has not been documented until now. In this article, we present the case of a 22-year-old previously healthy female patient who contracted COVID-19 complicated by an erythroedema on the back of the right hand, extending notably to the forearm on the 10th day of the infection. Skin biopsy revealed dermal and septal hypodermal fibrosis with a mild lymphocytic interstitial infiltrate in the dermis consistent with morphea. Treatment with low-dose corticosteroids was started, and regular follow-up was established. An isolated recurrence of cutaneous symptoms was observed after the first COVID-19 vaccination (Sputnik V) administered five months after the initial infection, with spontaneous regression in 10 days. This clinical evolution underscores the importance of a comprehensive understanding of dermatological manifestations in COVID-19, particularly in the context of post-infection vaccination.

## Introduction

Coronavirus disease 2019 (COVID-19) was initially considered a disease of the lung and the gastrointestinal tract but also shows many atypical presentations associated with this disease [1]. The dermatological manifestations associated with COVID-19 infection encompass either the exacerbation of pre-existing dermatoses such as eczema and psoriasis or the emergence of novel cutaneous manifestations, some of which are specific to this viral infection, contributing to the distinctive nature of this pathology (e.g., vasculitis, livedo, exanthema) [2]. In this context, we present a case, marking the first reported instance of linear morphea manifesting with a severe acute respiratory syndrome coronavirus 2 (SARS-CoV-2) virus infection, with a notable recurrence following vaccination. 

The dermatological implications of COVID-19 have increasingly garnered attention due to their diverse and evolving nature [3]. While previous studies have identified a spectrum of skin-related responses to the virus, the occurrence of morphea in the context of SARS-CoV-2 infection remains unexplored. Morphea, a rare skin disorder characterized by localized areas of thickened and sclerotic skin, typically presents as circumscribed plaques. The linear variant, however, manifests as linear bands of sclerosis affecting the extremities that can lead to cosmetic and functional disability (limitation of articulation movement) [4]. Its association with COVID-19 has not been documented until now.

This case report demonstrates linear morphea that emerged in the course of SARS-CoV-2 infection, shedding light on a previously unrecognized dermatological sequela of COVID-19, followed by recurrence during vaccination. The case presents an intriguing aspect of the reactivation of morphea following vaccination against the virus. The convergence of these two events prompts a thorough examination of the potential relationship between SARS-CoV-2, vaccination, and the recurrence of morphea, unraveling novel dimensions in our understanding of the intricate interplay between viral infections, immunization, and dermatological manifestations. We aim to contribute valuable insights to the expanding body of knowledge surrounding COVID-19-related dermatological manifestations, emphasizing the need for heightened clinical awareness and comprehensive investigations in the field.

## Case presentation

In January 2021, a 22-year-old previously healthy female patient contracted COVID-19. She initially presented with common symptoms of fatigue, fever, cough, and diarrhea. Diagnosis and clearing of infection were confirmed through PCR tests done, respectively, on the third and 21st days from symptom onset. The patient's condition did not require hospitalization.

**Figure 1:**
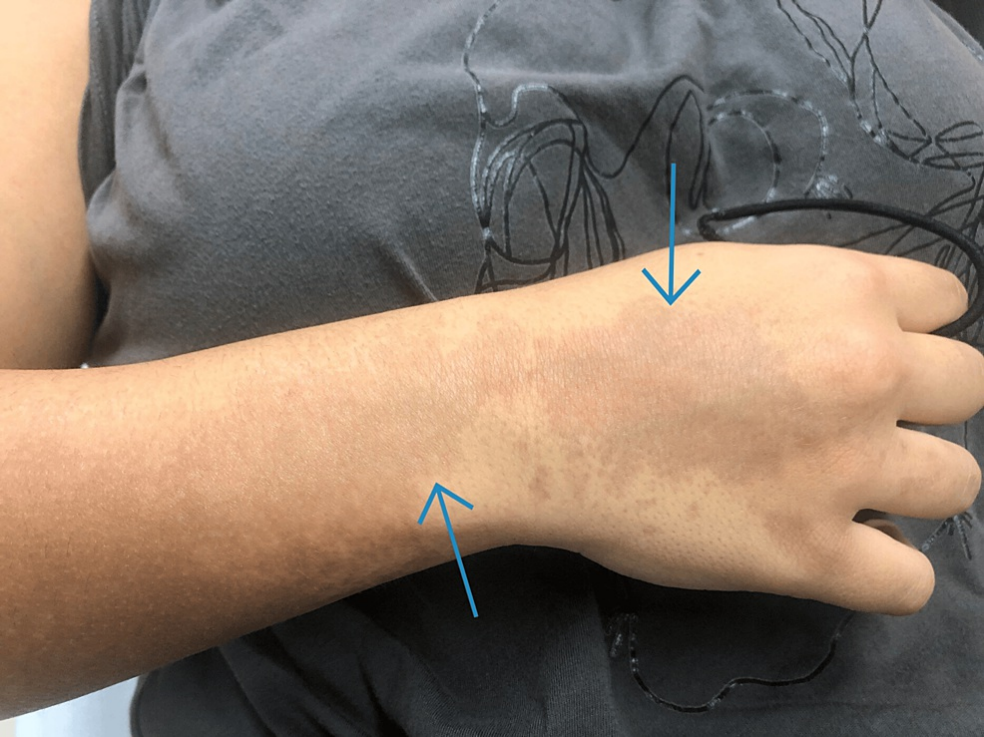
Initial patient presentation with a scarlet redness, unusual skin shine, and noticeable infiltration in the upper extremity skin

Approximately 10 days from the onset of the presenting symptoms of the infection, the patient developed erythroedema on the back of the right hand, extending to the right forearm. Clinical examination of the patient showed that the affected area exhibited a scarlet redness, unusual skin shine, and noticeable infiltration (Figure 1), while general examination yielded no significant abnormalities elsewhere.

**Figure 2:**
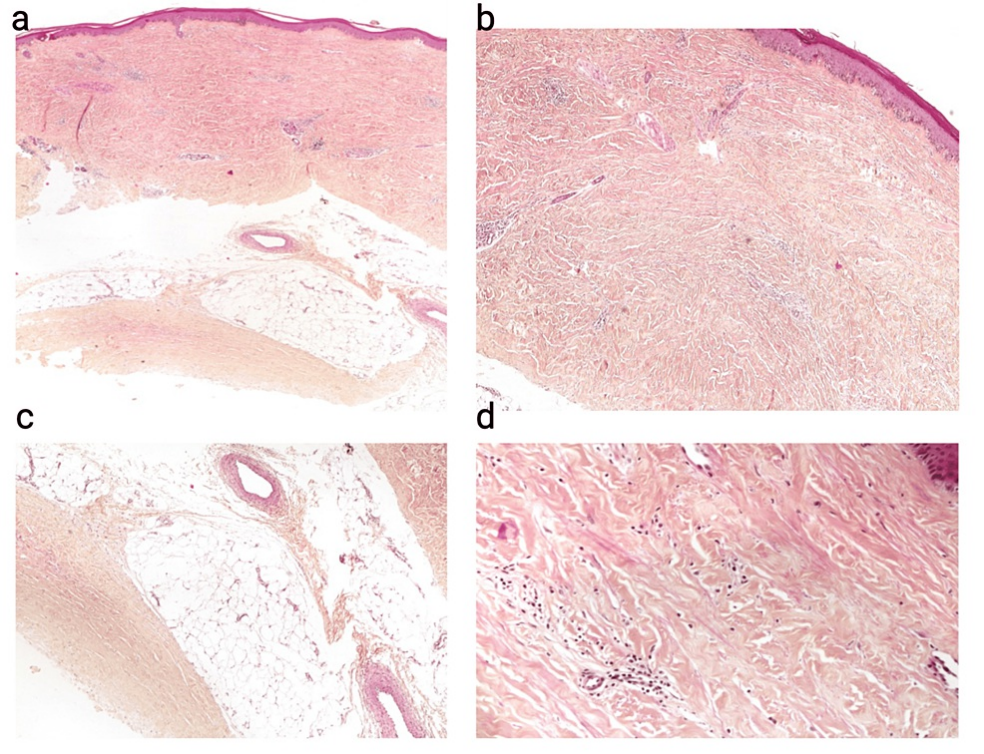
Skin biopsy taken from the forearm. Hematoxylin and eosin-stained section at ×40 (panel a), ×100 (panel b), ×200 (panel c), and ×400 (panel d) showing extensive dermal and septal hypodermal fibrosis with entrapment of the eccrine glands high in the dermis and a mild superficial and deep perivascular lymphocytic infiltrate with scattered plasma cells

Routine blood tests, including complete blood count (CBC), C-reactive protein (CRP), electrolytes, creatinine, urea, liver function tests, and thyroid stimulation hormone (TSH), were done, and all were negative or normal complementary investigatory blood tests including serologies for Epstein-Barr virus (EBV), *Cytomegalovirus* (CMV) and toxoplasmosis, and immunological markers (ANA, anti-DNA, anti-Sm antibodies, RNP, anticentromere antibodies, and anti-Scl 70 antibodies). All gave negative results. Furthermore, imaging and radiological examination of the affected areas were done. Standard X-ray and MRI with gadolinium showed a cloudy-appearing right olecranon with minimal liquid infiltration of the subcutaneous soft tissue. Finally, and in the light of all the negative aforementioned workup, a skin biopsy was done revealing dermal and septal hypodermal fibrosis with entrapment of the eccrine glands high in the dermis and a mild superficial and deep perivascular lymphocytic infiltrate with scattered plasma cells. The overlying epidermis exhibits mild hyperkeratosis, acanthosis, and basal keratinocyte pigmentation (Figure 2). Direct immunofluorescence studies were negative.

After excluding other differential diagnoses and based on the skin biopsy, imaging, and other laboratory results, the diagnosis of morphea was established, and a low-dose oral corticosteroid therapy (0.6 mg of betamethasone/day) was initiated after no response to topical corticosteroids used for four weeks. Clinical assessment of the patient three weeks later showed marked clinical symptom regression with complete clinical disease remission. Based on clinical assessment, oral corticosteroids were stopped four weeks after their initiation, and regular follow-up showed no relapse of the disease in the same previously affected area or elsewhere on the skin of the same patient.

However, five months later, the patient took the Sputnik V vaccine which put her into relapse: an isolated recurrence of cutaneous symptoms occurred with evident spontaneous regression in 10 days. Follow-up skin examinations at 10 and 24 months revealed complete remission from morphea, leaving behind a linear residual pigmentation of the previously affected area. Thus, this clinical scenario underscores the importance of a comprehensive understanding of dermatological manifestations in COVID-19, particularly in the context of post-infection vaccination.

## Discussion

The patient presented with linear morphea manifested during infection with SARS-CoV-2, with reactivation of cutaneous symptoms following the first COVID-19 vaccination. No systemic involvement of scleroderma was observed. This dermatosis is classified as plaque morphea, generalized morphea, linear scleroderma, and deep morphea. Among the reported cases associated with COVID-19, all were forms of generalized morphea [5]. The involvement observed in our case was a rather monomeric presentation, in contrast to the diffuse pansclerotic, adding uniqueness to the reported case. The immunological assessment yielded negative results.

In cases linking morphea to COVID-19, the first half occurred after vaccination while the other half after infection [6]. Regarding the reactivation of morphea after vaccination, vaccines such as diphtheria-tetanus-pertussis (DTP), hepatitis B, bacillus Calmette-Guérin (BCG), and pneumococcal have been reported to be associated with morphea. However, most of these conditions occurred locally at the vaccine injection sites, rather than presenting in more diffuse forms [7].

Morphea has been described in autoimmune diseases such as systemic lupus erythematosus, vitiligo, alopecia areata, rheumatoid arthritis, and autoimmune thyroiditis. It may be linked to various environmental factors, including trauma, radiation, medications, infections, and vaccines [8]. *Borrelia burgdorferi* is the infectious pathogen most associated with morphea [8]. Other infections with a probable association include hepatitis B virus, hepatitis C virus, *Cytomegalovirus*, toxoplasmosis, *Helicobacter pylori*, and human endogenous retroviruses [8].

Regarding the pathogenesis, given the genetic similarities between the spike protein of the SARS-CoV-2 vaccine and human proteins, molecular mimicry and production of self-reactive lymphocytes may have contributed to inducing this autoimmune disease in a more widespread clinical phenotype. This can lead to the activation of chemokines, cytokines, and especially type I interferon, which plays a central role in the pathogenesis of morphea and systemic sclerosis, correlating with disease activity. Skin and soft tissue modifications observed in case of morphea and infection by different viruses (as well as related vaccines) can trigger vascular lesions through neo-intimal proliferation by the overproduction of profibrotic cytokines (such as TGF-beta, PDGF-alpha, and PDGF-beta). Thus, SARS-CoV-2 infection induces cutaneous vascular lesions with massive cytokine release, activation of adhesion molecules, and T cells, potentially contributing to connective tissue disorders [2,5-9].

## Conclusions

This case report highlights unique linear morphea during a SARS-CoV-2 infection, with subsequent reactivation post-COVID-19 vaccination. This contrasts with the typical reported generalized morphea in COVID-19. Morphea is associated with autoimmune, environmental, and infectious factors, particularly *Borrelia burgdorferi*. Findings contribute to understanding viral-vaccination-autoimmune interplay. Molecular mimicry, self-reactive lymphocytes, and cytokine activation, especially type I interferon, may induce morphea. Overproduced profibrotic cytokines in vascular lesions may connect cutaneous manifestations to broader tissue disorders. Continued vigilance and research into COVID-19-related dermatological sequelae, especially post-vaccination, are crucial for comprehensive clinical management and understanding of evolving manifestations.
